# Mechanical Cues Regulate Histone Modifications and Cell Behavior

**DOI:** 10.1155/2022/9179111

**Published:** 2022-05-11

**Authors:** Buwei Hu, Dandan Zhou, Haoming Wang, Ning Hu, Weikang Zhao

**Affiliations:** ^1^Department of Orthopedics, The First Affiliated Hospital of Chongqing Medical University, Chongqing 40042, China; ^2^Zhejiang Provincial Laboratory of Life Sciences and Biomedicine, School of Life Sciences, Westlake University, Hangzhou, Zhejiang 310024, China; ^3^Department of Gastroenterology, The People's Hospital of Jiulongpo District, Chongqing 400050, China; ^4^Department of Orthopedics, Chongqing University Three Gorges Hospital, Chongqing 404100, China

## Abstract

Change of biophysical factors in tissue microenvironment is an important step in a chronic disease development process. A mechanical and biochemical factor from cell living microniche can regulate cell epigenetic decoration and, therefore, further induce change of gene expression. In this review, we will emphasize the mechanism that biophysical microenvironment manipulates cell behavior including gene expression and protein decoration, through modifying histone amino acid residue modification. The influence given by different mechanical forces, including mechanical stretch, substrate surface stiffness, and shear stress, on cell fate and behavior during chronic disease development including tumorigenesis will also be teased out. Overall, the recent work summarized in this review culminates on the hypothesis that a mechanical factor stimulates the modification on histone which could facilitate disease detection and potential therapeutic target.

## 1. Introduction

In the development of chronic disease, cells are exposed to many stimuli that require distinct response mechanisms to injury and tissue repair. During these processes, cells often exhibit extraordinary cell behavior and activities under change of regular living microenvironment. Cell behavior includes cytoskeleton reorganization, protein activity regulation, gene expression pattern change, and management of posttranscriptional regulation [1–3]. Posttranscriptional regulation requires the recruitment of microRNA and long noncoding RNA (lncRNA), both of them are involved in chronic disease development [4, 5].

Chronic disease is a long-term tissue injury process, resulting in microenvironmental cues that can manipulate cell status. Elegant control of cell behavior is important to reduce the impact given by chronic disease [6]. Furthermore, the change of cell living microenvironment could lead to both biochemical rewiring and physical reshaping of all cellular compartments on both short and long timescales.

**Figure 1:**
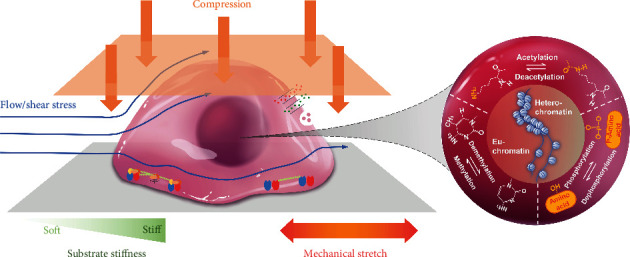
Mechanical cues regulate histone modifications to modulate cell fate. Various mechanical stimuli, including substrate stiffness, mechanical stretch, topological substrate, shear stress, compression, and squeezing, modulate histone modifications, which regulate chromatin reorganization and cell plasticity.

Suffering from a long-term tissue injury process, the mechanical factors in the cell microenvironment, including compression, substrate stiffness, stretch, and shear stress, have changed and influenced cell properties [7, 8]. These forces can induce nuclear deformation or activate biochemical pathways and thus regulate the epigenetic state in a mechanotransduction-dependent manner (shown in Figure 1) [9]. Additionally, the chemical and physical signals induced by mechanical stimuli are shaping the cell gene expression pattern based on chromatin remodeling enzymes and the transcriptional machinery [10]. At the same time, epigenetic regulation could manipulate chromosome structure including heterochromatin with limited transcription activity and less condensed, transcription active euchromatin regions. This process is achieved based on the decoration on amino acid residues of histone and their variants, including methylation and acetylation [11, 12]. The acetylation/deacetylation of histones controls global chromatin structure and influences the gene active transcription area [13]. Methylation/demethylation of DNA is also involved in modulating chromatin condensation, and this process is highly correlated with cell metabolism and behavior [14]. Overall, these epigenetic modifications could be caused by the change of mechanical microenvironment and impact cell gene expression and, therefore, further promote chronic disease development and set barriers to patient recovery. In this review, we will discuss how various mechanical cues modulate cell behavior based on histone modification.

## 2. Extracellular Matrix Stiffness

Many studies have shown that the tissue fibrosis escalates the stiffness of the extracellular matrix (ECM) playing an influential role in tumor development and atherosclerosis [1, 3]. The ECM of tumor consists of collagen I, elastin, fibronectin, hyaluronan, and sulfated glycosaminoglycan. The cancer-associated fibroblast (CAF) secretes the majority of ECM components and facilitates tumor microenvironment construction [1]. Increasing ECM stiffness further manages fibroblast ECM secretion and macrophage cytokine production, induces cancer cell proliferation, and promotes metastasis [2].

During lung cancer development, the epigenetic modification on an oncogene promoter facilitates the construction of mechanical microenvironment. Under stimulation, the Yes-associated protein (YAP) aggregates in the nucleus of the human lung adenocarcinoma cell (H1299), which is induced by RASFF1A promoter methylation and promotes prolyl 4-hydroxylase alpha-2 (P4HA2) expression. The expression of P4HA2 further induces collagen production and makes the substrate stiffer [15]. Similarly, in gastric cancer, YAP reciprocally interacts with DNA methylation inhibitors GRHL2, TET2, and KMT2A to prevent YAP promoter methylation, causing oncogenic activation of YAP [16]. On the soft substrate, Liu et al. reported CDC42 accumulation in the cell nucleus and promote Tet2 expression, which demethylates the promoter of P21 and P27 to shift the cancer cell to dormancy status, which helps cancer cells overcome harsh conditions [17]. Under similar conditions, tumor-repopulating cells (TRC) preserve their plasticity through downregulating G7a to reduce histone3 lysine residue 9 (H3K9) methylation and result in Sox2 expression [18]. Based on these mechanisms, cancer cells can preserve their plasticity and halt their proliferation progress until reaching appropriate position for tumor regeneration.

Not only cancer but also tissue fibrosis process happening on different organs can manipulate cell epigenetic change and result in organ pathological damage. The lung epithelial cell during idiopathic pulmonary fibrosis escalates desmoplakin (DSP) expression due to demethylation of its promoter. Accumulation of DSP increases cell-cell adhesion force and promotes lung fibrosis [19]. Furthermore, H3K9me decoration maintains lung fibroblast under activation status to produce collagen during lung fibrosis. The G9a and CBX5 histone methyltransferase is activated in a stiffer matrix and mediates histone methylation to induce ECM protein overexpression [20]. Similar to lung fibroblast, stiffness of the substrate also can regulate vascular smooth muscle cell (VSMC) epigenetic modification. On higher stiffness ECM, VSMC downregulates the expression of DMNT1 and reduces the general methylation status. Xie et al. reported that DMNT1 can manipulate the promoter of SM22a and SMA, both of them were involved in SMC functional contractility and induce vascular disease [21]. Altogether, these studies demonstrate that matrix stiffness can modulate the epigenome to facilitate disease development and impact the cell gene expression pattern (Table 1).

## 3. Mechanical Strain

Mechanical stretch is another biophysical factor that can influence cell gene expression, disease development, and tissue regeneration. Cells in the vascular wall and lung tissue are permanently exposed to cyclic or stain mechanical stretch. Under high blood pressure, cyclic strain on vascular cell increases vascular wall thickness and facilitates reactive oxygen species (ROS) generation [25]. Similar to the vessel, cyclic stretch in the lung stimulates local fibroblast and macrophage change biochemical microenvironment [26]. In addition to the influence on cell physiological status, mechanical strain is also involved in the tissue regeneration process.

Cyclic strain has already shown potential to mediate tissue regeneration including the osteogenesis process [27]. With strain stimulation, the epigenetic status of cell is changed and regulates cell differentiation. DNA methylation is a common epigenetic modification that is modulated during cell differentiation. Human adipose tissue multipotential stromal cell (hAT-MSCs) promote osteogenic induction by cyclic stretch-induced DNA demethylation on the CpG of the GNAS gene[28].

In addition to DNA methylation, the modification of histones is another epigenetic mechanism that is influenced by mechanical stretch. HDACs regulate histone acetylation, thereby modulating the chromatin state and gene expression. In lung endothelial dysfunction, cyclic stretch induces HDAC6 activation and is downregulated in endothelial cells (EC) and results in a-tubulin deacetylation[29]. However, instead of changes in tubulin modification, HDAC4 translocation from the cytoplasm to the nucleus in murine chondrocytes after stretch repressed RUNX-2 expression [30]. In chondrocytes, cyclic stretch also stimulates the expression of ADAMTS-5 which could induce osteoarthritis. The inhibitor of HDAC could reduce the expression of ADAMTS-5 and provide a potential solution for osteoarthritis [31]. Although numerous studies have shown that mechanical stretch can regulate HDAC expression, further research into the mechanism of this HDAC regulation is still required.

Recent studies demonstrating the mechanism of posttranscriptional regulation, including microRNA and lncRNA, in the cell under strain can provide some valuable insight into the direction for future research. Interestingly, in human chondrocytes, miR-365 levels decreased after stretch, which led to a downregulation of ACAN gene expression and cartilage degeneration [32]. Also, after stretch, HPMEC expressed lncRNA mainly enriched in response to hypoxia and inflammatory response. This result provides a research clue on ventilator-induced lung injury [33]. Therefore, it may be possible that mechanical strain induces specific microRNAs in different cell types to influence the expression of histone modification marks and as a result mediate the epigenetic state and cell gene expression.

Cell proliferation is also involved in disease development progress. Cell division could be facilitated through histone 3 phosphorylation (H3P), which can produce a loosen chromatin region for active gene expression. In response to mechanical strain, the activation of the Piezo1 ion channel induced H3P and promoted cell proliferation [34]. Similarly, in neointimal hyperplasia, cell proliferation is facilitated by vascular stretch, and microRNA-33 expression repressed to facilitate cell division [35, 36].

Taken together, these studies demonstrate that mechanical stretch regulates DNA methylation, histone phosphorylation, acetylation, and methylation to modulate cell behavior. Furthermore, both static and cyclic strains can influence HDAC expression directly or through miRNA and lncRNA regulation. Currently, most research has been focused on respiratory and cardiovascular disease. Yet, how mechanical forces, such as compression and strain, affect epigenetic mechanisms in cancer remains unclear. A summary of the effects of mechanical stretch on the epigenetic state and cell behavior discussed in this section can be found in Table 2.

## 4. Shear Stress

Shear stress can also directly regulate histone-modifying enzymes, which play a critical role in cardiovascular disease development, including atherosclerosis. The increase in shear stress mediates endothelial cell gene expression and regulates the local immune microenvironment [7]. The spreading of the nucleus caused by cytoskeleton reorganization led to the accommodation of more signaling molecules and transcription factors. Previous researches have illustrated that HDACs are significantly involved in regulating hemodynamic-induced EC function and dysfunction (Table 3). Bazou et al. [37] reported that the interstitial flow increased HDAC1 phosphorylation which promotes endothelial morphogenesis and matrix metalloproteinase-14 (MMP14) expression. Activated vascular ECs showed higher growth and migration capabilities, which could contribute to angiogenesis. The involvement of HDAC6 facilitates endothelial cell migration and could potentially induce an atherosclerosis lesion [38]. In addition, low fluid shear stress and EGR-KLF2 cooperatively regulated the transcriptional expression of anticoagulant thrombomodulin (TM) [39]. ERG binding to the TM promoter recruits p300 and induces H3K27 acetylation, which directly drives the exposure of the TM promoter region further inducing TM expression in ECs.

On the contrary, high shear stress (65-85 dyn/cm^2^) also could affect cell plasticity, possibly even leading to disease. For example, through attenuating the HDAC5 nuclear export, high laminar shear stress modulates the interaction between HDAC5 and KLF2 in HUVEC (human umbilical vein cell). Furthermore, pulsatile shear stress (PS) is believed to prevent atherosclerosis by maintaining cell homeostasis [40, 41]. The key regulator KLF4 can regulate PS-mediated H3K27ac, which contributes to the Ca^2+^-dependent eNOS activation and EC homeostasis [42].

A recent study found that shear stress can modulate the expression of the polycomb methyltransferase EZH2 [43]. Xu et al. [44] reported that 20 dynes/cm^2^ laminar shear stress could decrease the expression of EZH2 through the mechanosensitive microRNA miR101 in ECs. The downregulation of EZH2 expression concurrently further decreases methylation on H3K27, which limits endothelial inflammatory factor expression. This research has revealed that in atheroprotective flow, the reduction of H3K27me3 is the potential histone-based mechanism to augment anti-inflammatory gene expression in the endothelium. Moreover, through EZH2, high uniform shear stress also could induce cell quiescence through limiting the expression of cell cycle-related genes.

In addition to histone modifications, shear forces can also modulate cell behavior through DNA modifications. Monocyte adhesion on the HUVEC surface was decreased when the DNMT1 inhibitor 5-Aza was administered, indicating that DNMT1 regulated the inflammation of ECs. Moreover, arteriogenic capacity and arterial vascular remodeling also can be regulated by DNMT1. Heuslein et al. [41] determined that nonreversed flow collateral segments exhibited general *in vivo* DNA hypermethylation. Furthermore, ECs exposed to the nonreversed waveform escalate DNMT1 expression, which induces hypermethylation of the promoters of significantly regulated genes and a DNMT1-dependent reduction in proatherogenic monocyte adhesion [45].

Other mechanical forces, such as pressure, can also regulate histone modifiers to influence cell function. For example, mechanical forces play a fundamental role in regulating cartilage morphogenesis and maintenance. HDAC4 is a transcriptional regulatory protein involved in regulating the cell cycle, cartilage development, and endochondral ossification [46]. Cheleschi et al. demonstrated the beneficial role of cyclic hydrostatic pressure, suggesting the involvement and regulation of HDAC4 activation in osteoarthritis chondrocytes [47]. Moreover, some diseases lead to increased pressure in local tissues that alter the epigenetic state. For example, in the fetal lamb model, persistent pulmonary hypertension reorganized the epigenetic characteristics in pulmonary artery ECs, especially the nitric oxide synthase gene [48]. This process was mediated through a decrease in H4K12ac and an increase in H3K9me3, which are active and repressive marks that were found at the binding site of Sp1 in the eNOS promoter region, respectively. In a retinal ischemia-reperfusion (IR) injury mouse model, elevated intraocular pressure activated glial cells as a result of increased HDAC2 activity [49].

Altogether, findings from these studies suggest that methylation and acetylation of histones can be modulated by shear stress and therefore affect cell plasticity, including stem cell differentiation, cellular transitions (e.g., EMT and EndMT), and EC function. In some cases, high shear stress was able to induce stem cell differentiation and protect ECs from inflammation whereas low or oscillating shear stress induced EC inflammation and promoted arteriosclerosis. Furthermore, DNA methylation can also be regulated by fluid shear forces to affect cellular behavior. Apart from fluid shear force, local high pressure under pathological and physiological conditions also plays a role in regulating histone modifiers that affect cellular plasticity.

## 5. Nanotopography and Nanostructure

Surface nanotopography, structure, and pattern are important factors to influence cell behavior and differentiation, and a summary of the effects can be found in Table 4. Under pathological condition, the tumor will have a special surface pattern, which can be used as a criterion to give diagnosis [50]. In the tissue engineering area, material nanotopography influences cell differentiation and reprogramming had been widely discussed. In bone regeneration research, TiO_2_ is a widely used material to support osteogenesis. Based on the previous research, a TiO_2_ nanotube array was found to have the ability to promote hASC osteogenic differentiation. On the nanotube array, HDAC expression was downregulated and induces histone acetylation and promotes KDM3E gene expression, which facilitates bone regeneration [51]. Also, through mimicking the physiological tissue constraint, a three-dimensional synthesizing matrix promotes fibroblast rejuvenation through facilitating chromatin decondensation [52]. These phenomena illustrated the importance on nanotopography and structure in tissue regeneration and cell reprogramming.

Microgroove is a fundamental model used to investigate influence given by surface topography. On microgrooves, the reprogramming efficiency of fibroblast will be influenced through histone modification. In IPSC reprogramming, microgroove could reduce HDAC expression and increase the H3 methytransferase subunit, WDR5, and expression. This process increases histone acetylation and H3K4 methylation and facilitates the cell to perform stemness [53]. Under a similar mechanism, it was also found that microgroove can facilitate induced dopaminergic neuron reprogramming from the fibroblast [54]. Related researches also had been done on the stem cell. In BMSC, it was also found that due to the nuclear shape change given by microgrooves, the force applied on the nucleus deformed the lamin A/C around the cell nucleus, which could be stimulated and modulate the epigenetic modification, including HDAC activity and histone acetylation [55]. The cardiac progenitor cell also found that under similar stimulation, histone acetylation is upregulated and promotes mycardin and Tbx5 gene expression to promote cardiovascular differentiation [56]. Similar with microgrooves, the micropattern also could regulate cell differentiation and tissue regeneration. It was found that the micropattern which decreases the cell nuclear area downregulates H3K9ac and increases H3K9me3. This process increases MSC proangiogenic activity and promotes angiogenesis [57].

For the cancer cell, current research is more focused on the impact given by the micropattern. In breast cancer, micropatterning can facilitate cancer cell gain stemness through increasing H3K9ac and prohibiting H3K27me3. This result also found mouse embryonic fibroblast [58]. Similarly, micropattern curvature can manipulate histone methylation and increase tumor cell heterogenesis [59]. However, microgrooves can influence cancer cell proliferation and migration and may be involved in metastasis [60–62]. The research on how this factor impacts cancer cell epigenetic modification is still under investigation. The research on how the nanotopographical pattern in the tumor influences cancer cell epigenetic activity could provide another insight into cancer therapy development.

## 6. Conclusions and Future Directions

Chronic disease change of tissue mechanical microenvironment continuously stimulates local or recruited cell populations via mechanotransduction pathways. In arteriovenous malformation caused by hereditary hemorrhagic telangiectasia, shear stress stimulates type I/II serine/threonine kinase receptor (ALKs) on the endothelial cell surface and regulates interaction between the receptor and bone morphogenetic proteins 9 and 10 (BMP9 and BMP10). This cascade can regulate heat development though change expression of SMAD protein [63]. Also, change of matrix stiffness during atherosclerosis development regulates ECs through the RhoA/ROCK pathway and MAPK pathway [64]. Similarly, ECM stiffness regulates epidermal cell proliferation, migration, and differentiation based on the Rho/ROCK pathway through integrin stimulation [65]. Additionally, the mechanical properties of cell, including cell stiffness, could become potential therapeutic target for cancer chemotherapy and immune therapy [66, 67].

Different mechanical cues can initiate distinct epigenetic modifications, even on the same histone, such as HDAC family members which have varied responses to shear stress and mechanical stretch, and modifications of H3K9 in response to matrix stiffness and shear stress are distinctive as well. All of these processes involve numerous complex systems which include not only the cytoskeleton-nuclear skeleton-chromatin pathway but also mechanical-chemical coupling. Current work has put most attention on investigating the epigenetic modification under pathological mechanical stimulation. The knowledge in this area provides another opportunities to develop more effective therapies for tissue repair and cancer treatment. However, the relationship between the cell signaling pathway and change of epigenetic status still needs further investigation.

Interestingly, the relationship between mechanical microenvironment factors and epigenetic modification for immune cells including macrophages still remains blank. It is known that macrophages can sense stiffness, shear stress, cyclic stretch, and microgroove pattern and induce macrophage polarization and cytokine expression [68, 69]. Also, epigenetic modification is related to cytokine secretion and macrophage biochemical microenvironment construction [70, 71]. However, how mechanical factors influence macrophage epigenetic modification remains to be elucidated. The mechanism that pathological biophysical microenvironment regulates macrophage histone modification still needs further investigation.

Although a large number of studies have provided insights into the mechanism by which mechanical cues affect histone modifications and regulate cell gene expression, some questions still remain unclear. Mechanical stimuli, such as cyclic stretch, can induce more than one epigenetic modification process. However, the priority of types of epigenetic modification in different cell types and which mechanical stimuli have a dominant impact on different diseases still need further investigation. Similarly, whether mechanical-chemical coupling is involved during the modulation of histone modifications should be further examined. All of these questions require further investigation and will provide incisive understanding on pathological influence given by mechanical microenvironment change in disease development and provide new therapeutic targets.

## Figures and Tables

**Table 1:** Summary of the effects of matrix stiffness on histone modifications in cell plasticity.

Cell type	Stiffness tested	Epigenetic modification	Function	Ref.
H1299	0.5-25 kPa	DNA methylation	RASSF1A promoter methylation	[15]
MDA-MB-231	0.5-50 kPa	Histone demethylation	ECM stiffness regulates histone demethylase JMJD1a nuclear aggregation	[22]
Fibroblast	0.2-64 kPa	H3K9me2/3	Stiff matrices increase global H3K9me2/3	[20]
A549	1-20 kPa	DNA hypomethylation/demethylation	Observed DNA hypomethylation/demethylation under stiff matrix conditions	[19]
MEFs	0.5-100 kPa	Histone deacetylation	HDAC4 expression increased	[23]
HUASMCs	2.16-16.75 kPa	DNA methylation	Substrate stiffening downregulation of DNMT1 and DNA methylation	[21]
AGS	0.5-7 kPa	DNA methylation	Stiffer substrate induces reduction in global methylation	[16]
TRC	50-1050 Pa	DNA methylation	Soft surface demethylates p21 and 27 promoters	[17]
DLD-1	0.4-25.6 kPa	H3K4me3	Reduction of H3K4me3 on softer matrices	[19]
TRC	50-1050 Pa	H3K9me	Softening of the matrix reduces H3K9 methylation	[18]
MEF	0.5-20 kPa	Microtubule acetylation	Soft matrix modulates microtubule acetylation	[24]

MSCs: mesenchymal stem cells; HSCs: hepatic stellate cells; hMSCs: human mesenchymal stem cells; MCF10A: mammary epithelial cells; A549: human alveolar epithelial adenocarcinoma cells; DLD-1: colorectal adenocarcinoma cells; MEFs: mouse embryonic fibroblasts; HUASMCs: human umbilical artery smooth muscle cell; H1299: human lung adenocarcinoma cell; MDA-MB-231: breast cancer cell; AGS: human gastric cancer cell; TRC: tumor repopulating cell; HDAC: histone deacetylase.

**Table 2:** Summary or examples of the effects of mechanical stretch on histone modifications.

Cell type	Stretch parameters	Epigenetic modification	Function	Ref
SMC	Cyclic strain, 10% elongation, 1.25 Hz	MicroRNA 33	Stretch represses miR33 expression	[35]
Epithelial cell	10-33% elongation	Histone H3P	Promoted cell proliferation	[34]
hAT-MSC	2.5-15% elongation, 1 Hz	DNA demethylation	GNAS CpG demethylation	[28]
HC	10% elongation, 0.5 Hz	miR-365 reduced HDAC4	Cartilage degeneration, downregulated ACAN	[32]
MLVEC	Cyclic stretch, 20% elongation, 30 cycles/min	HDAC6	Activated HDAC6 induces deacetylation of a-tubulin	[29]
HC	Cyclic stretch, 10% elongation, 0.5 Hz	HDAC	HDAC inhibitor downregulates RUNX-2, ADAMTS-5, and MMP-3 expression	[31]
HPMEC	Cyclic stretch, 20% elongation	Differentially expressed lncRNA (DEL)	lncRNA might regulate inflammation and fibrosis	[33]
C2C12	Cyclic stretch, 5% elongation	MicroRNA 146a	Increasing miR-146 expression which downregulates Numb	[36]
MC	0.25 Hz 6% elongation	HDAC4, histone 3 deacetylation	Promoted collagen II and repressed RUNX2 expression	[30]

MSC: mesenchymal stem cells; hAT-MSC: human adipose tissue multipotential stromal cell; SMC: smooth muscle cell; HC: human chondrocyte; MLVEC: mouse lung vascular endothelial cell; HPMEC: human pulmonary microvascular endothelial cell; C2C12: mouse myoblast; MC: murine chondrocyte.

**Table 3:** Examples of shear stress-regulated histone modifications.

Cell type	Shear stress	Histone/gene	Function	Ref.
VSMC	15 dynes/cm^2^	HDAC6	Shear stress increases HDAC6 and 5downregulates acetylated tubulin	[38]
ECs	Interstitial flow	HDAC1	Increased morphogenesis, MMP14, and angiogenesis	[37]
HUVECs	5 dynes/cm^2^ shear stress	H3K4Me3, H3K27Ac	Increased thrombomodulin expression	[39]
HUVECs	12 ± 4 dynes/cm^2^ pulsatile shear stress	H3K27ac	Ca^2+^-dependent eNOS activation, EC homeostasis	[42]
ECs	20 dynes/cm^2^ laminar shear stress	EZH2	Deceased cell cycle and promoted quiescence	[43]
HUVECs	12 dynes/cm^2^ laminar flow	H3K27me3	Confer an anti-inflammatory response	[44]
HUVECs	15-30 dynes/cm^2^ shear stress	DNMT1	Exhibited DNA hypermethylation	[45]
Chondrocytes	0.5 MPa hydrostatic pressure	HDAC4	Downregulate MMP-13, ADAMTS-5, and HDAC4	[47]
ECs	Pulmonary hypertension	H4K12ac, H3K9me3	Decreased expression of eNOS	[48]
GC	Intraocular pressure	HDACs	Enhanced glial activation following IR injury	[49]

ECs: endothelial cells; ESCs: embryonic stem cells; HUVECs: human umbilical vein cells; VSMC: vascular smooth muscle cell; GC: glial cell.

**Table 4:** Examples of nanotopography-regulated histone modifications.

Cell type	Nanotopography	Epigenetic modification	Function	Ref
hASC	Nanotube array	HDAC	Promote osteogenic differentiation	[51]
Fibroblast	Microgroove	HDAC	Facilitate IPSC reprogramming	[53]
BMSC	Microgroove	HDAC	Not tested in research	[55]
Fibroblast	Microgroove	H3K4me3	Promote induced dopaminergic neuron reprogramming	[54]
MSC	Micropatterning	H3K9ac, H3K9me3, H3K36me3	Increase MSC proangiogenic activity and increase angiogenesis	[57]
MCF7	Micropatterning	H3K9ac, H3K4me3, K27me3	Induce transcriptome change and nuclear reprogramming, increase cell stemness	[58]
Fibroblast	Tissue 3D constraints	HDAC and H3K9ac	Increase cell sensitivity to mechanostimulation, promote essential processes for cell rejuvenation	[52]
MIC	Micropatterning	H3K4me2 and H3K9ac	Upregulate PRDM14 gene and influence melanoma heterogeneity	[59]
Cardiac progenitors	Microgroove	Histone acetylation	Increase myocardin, tbx5, and mef2c expression	[56]

hASC: human adipose tissue-derived stem cell; BMSC: bone marrow mesenchymal stem cells; MIC: malignant melanoma-initiating cell.

## Data Availability

The data used to support the findings of this study are available from the corresponding author upon request.

## References

[1] Kalli M., Stylianopoulos T. (2018). Defining the role of solid stress and matrix stiffness in cancer cell proliferation and metastasis. *Frontiers in Oncology*.

[2] Emon B., Bauer J., Jain Y., Jung B., Saif T. (2018). Biophysics of tumor microenvironment and cancer metastasis - a mini review. *Computational and Structural Biotechnology Journal*.

[3] Palombo C., Kozakova M. (2016). Arterial stiffness, atherosclerosis and cardiovascular risk: pathophysiologic mechanisms and emerging clinical indications. *Vascular Pharmacology*.

[4] He R.-Z., Luo D.-X., Mo Y.-Y. (2019). Emerging roles of lncRNAs in the post-transcriptional regulation in cancer. *Genes & Diseases*.

[5] Filipowicz W., Bhattacharyya S. N., Sonenberg N. (2008). Mechanisms of post-transcriptional regulation by microRNAs: are the answers in sight?. *Nature Reviews Genetics*.

[6] Bejarano L., Jordāo M. J. C., Joyce J. A. (2021). Therapeutic targeting of the tumor microenvironment. *Cancer Cell*.

[7] Yurdagul A., Finney A. C., Woolard Matthew D., Orr A. W. (2016). The arterial microenvironment: the where and why of atherosclerosis. *Biochemical Journal*.

[8] Liu Q., Luo Q., Ju Y., Song G. (2020). Role of the mechanical microenvironment in cancer development and progression. *Cancer Biology & Medicine*.

[9] Lv L. W., Tang Y. M., Zhang P., Liu Y. S., Bai X. S., Zhou Y. S. (2018). Biomaterial cues regulate epigenetic state and cell functions-a systematic review. *Tissue Engineering Part B-Reviews*.

[10] Uhler C., Shivashankar G. V. (2017). Chromosome intermingling: mechanical hotspots for genome regulation. *Trends in Cell Biology*.

[11] Allshire R. C., Madhani H. D. (2018). Ten principles of heterochromatin formation and function. *Nature Reviews Molecular Cell Biology*.

[12] Janssen A., Colmenares S. U., Karpen G. H. (2018). Heterochromatin: guardian of the genome. *Annual Review of Cell and Developmental Biology*.

[13] Narita T., Weinert B. T., Choudhary C. (2019). Author correction: functions and mechanisms of non-histone protein acetylation. *Nature Reviews Molecular Cell Biology*.

[14] Wu X. J., Zhang Y. (2017). TET-mediated active DNA demethylation: mechanism, function and beyond. *Nature Reviews Genetics*.

[15] Pankova D., Jiang Y., Chatzifrangkeskou M. (2019). RASSF1A controls tissue stiffness and cancer stem-like cells in lung adenocarcinoma. *The EMBO Journal*.

[16] Jang M., An J., Oh S. W. (2021). Matrix stiffness epigenetically regulates the oncogenic activation of the Yes-associated protein in gastric cancer. *Nature Biomedical Engineering*.

[17] Liu Y., Lv J., Liang X. (2018). Fibrin stiffness mediates dormancy of tumor-repopulating cells via a Cdc42-driven Tet2 epigenetic program. *Cancer Research*.

[18] Tan Y., Tajik A., Chen J. (2014). Matrix softness regulates plasticity of tumour-repopulating cells via H3K9 demethylation and Sox2 expression. *Nature Communications*.

[19] Qu J., Zhu L. Y., Zhou Z. J. (2018). Reversing mechanoinductive DSP expression by CRISPR/dCas9-mediated epigenome editing. *American Journal of Respiratory and Critical Care Medicine*.

[20] Ligresti G., Caporarello N., Meridew J. A. (2019). CBX5/G9a/H3K9me-mediated gene repression is essential to fibroblast activation during lung fibrosis. *Insight*.

[21] Xie S.-A., Zhang T., Wang J. (2018). Matrix stiffness determines the phenotype of vascular smooth muscle cell in vitro and in vivo: role of DNA methyltransferase 1. *Biomaterials*.

[22] Birukov K. G. (2009). Cyclic stretch, reactive oxygen species, and vascular remodeling. *Antioxidants & Redox Signaling*.

[23] Pugin J., Dunn-Siegrist I., Dufour J., Tissières P., Charles P. E., Comte R. (2008). Cyclic stretch of human lung cells induces an acidification and promotes bacterial growth. *American Journal of Respiratory Cell and Molecular Biology*.

[24] Gao J., Fu S., Zeng Z. (2016). Cyclic stretch promotes osteogenesis-related gene expression in osteoblast-like cells through a cofilin-associated mechanism. *Molecular Medicine Reports*.

[25] Vlaikou A. M., Kouroupis D., Sgourou A. (2017). Mechanical stress affects methylation pattern of GNAS isoforms and osteogenic differentiation of hAT-MSCs. *Biochimica et Biophysica Acta (BBA) - Molecular*.

[26] Wang Y., Liu Y.-J., Xu D.-F. (2021). DRD1 downregulation contributes to mechanical stretch-induced lung endothelial barrier dysfunction. *Theranostics*.

[27] Chen C., Wei X., Lv Z. (2016). Cyclic equibiaxial tensile strain alters gene expression of chondrocytes via histone deacetylase 4 shuttling. *PLoS One*.

[28] Saito T., Nishida K., Furumatsu T., Yoshida A., Ozawa M., Ozaki T. (2013). Histone deacetylase inhibitors suppress mechanical stress-induced expression of RUNX-2 and ADAMTS-5 through the inhibition of the MAPK signaling pathway in cultured human chondrocytes. *Osteoarthritis and Cartilage*.

[29] Zheng Q., Li X. X., Xiao L. (2019). MicroRNA-365 functions as a mechanosensitive microRNA to inhibit end plate chondrocyte degeneration by targeting histone deacetylase 4. *Bone*.

[30] Wang D., Dai C., Zhang X. (2021). Identification and functional analysis of long non-coding RNAs in human pulmonary microvascular endothelial cells subjected to cyclic stretch. *Frontiers in Physiology*.

[31] Gudipaty S. A., Lindblom J., Loftus P. D. (2017). Mechanical stretch triggers rapid epithelial cell division through Piezo1. *Nature*.

[32] Huang K., Bao H., Yan Z.-Q. (2017). MicroRNA-33 protects against neointimal hyperplasia induced by arterial mechanical stretch in the grafted vein. *Cardiovascular Research*.

[33] Bazou D., Ng M. R., Song J. W., Chin S. M., Maimon N., Munn L. L. (2016). Flow-induced HDAC1 phosphorylation and nuclear export in angiogenic sprouting. *Scientific Reports*.

[34] Wang Y. H., Yan Z. Q., Qi Y. X. (2010). Normal shear stress and vascular smooth muscle cells modulate migration of endothelial cells through histone deacetylase 6 activation and tubulin acetylation. *Annals of Biomedical Engineering*.

[35] Peghaire C., Dufton N. P., Lang M. (2019). The transcription factor ERG regulates a low shear stress-induced anti-thrombotic pathway in the microvasculature. *Nature Communications*.

[36] Ajami N. E., Gupta S., Maurya M. R. (2017). Systems biology analysis of longitudinal functional response of endothelial cells to shear stress. *Proceedings of the National Academy of Sciences of the United States of America*.

[37] Gongol B., Marin T., Zhang J. (2019). Shear stress regulation of miR-93 and miR-484 maturation through nucleolin. *Proceedings of the National Academy of Sciences of the United States of America*.

[38] He M., Huang T. S., Li S. (2019). Atheroprotective flow upregulates ITPR3 (inositol 1,4,5-trisphosphate receptor 3) in vascular endothelium via KLF4 (Krüppel-like factor 4)-mediated histone modifications. *Arteriosclerosis, Thrombosis, and Vascular Biology*.

[39] Maleszewska M., Vanchin B., Harmsen M. C., Krenning G. (2016). The decrease in histone methyltransferase EZH2 in response to fluid shear stress alters endothelial gene expression and promotes quiescence. *Angiogenesis*.

[40] Xu S., Xu Y., Yin M. (2018). Flow-dependent epigenetic regulation of IGFBP5 expression by H3K27me3 contributes to endothelial anti-inflammatory effects. *Theranostics*.

[41] Heuslein J. L., Gorick C. M., Song J., Price R. J. (2017). DNA methyltransferase 1-dependent DNA hypermethylation constrains arteriogenesis by augmenting shear stress set point. *Journal of the American Heart Association*.

[42] Yang X., Guan Y., Tian S., Wang Y., Sun K., Chen Q. (2016). Mechanical and IL-1*β* responsive miR-365 contributes to osteoarthritis development by targeting histone deacetylase 4. *International Journal of Molecular Sciences*.

[43] Cheleschi S., De Palma A., Pecorelli A. (2017). Hydrostatic pressure regulates microRNA expression levels in osteoarthritic chondrocyte cultures via the Wnt/*β*-catenin pathway. *International Journal of Molecular Sciences*.

[44] Ke X., Johnson H., Jing X. (2018). Persistent pulmonary hypertension alters the epigenetic characteristics of endothelial nitric oxide synthase gene in pulmonary artery endothelial cells in a fetal lamb model. *Physiological Genomics*.

[45] Sung M. S., Heo H., Eom G. H. (2019). HDAC2 regulates glial cell activation in ischemic mouse retina. *International Journal of Molecular Sciences*.

[46] Tanaka K., Toyoda H., Kadowaki S. (2008). Surface pattern classification by enhanced-magnification endoscopy for identifying early gastric cancers. *Gastrointestinal Endoscopy*.

[47] Lv L., Liu Y., Zhang P. (2018). The epigenetic mechanisms of nanotopography-guided osteogenic differentiation of mesenchymal stem cells via high-throughput transcriptome sequencing. *International Journal of Nanomedicine*.

[48] Roy B., Yuan L., Lee Y., Bharti A., Mitra A., Shivashankar G. V. (2020). Fibroblast rejuvenation by mechanical reprogramming and redifferentiation. *Proceedings of the National Academy of Sciences*.

[49] Downing T. L., Soto J., Morez C. (2013). Biophysical regulation of epigenetic state and cell reprogramming. *Nature Materials*.

[50] Yoo J., Noh M., Kim H., Jeon N. L., Kim B.-S., Kim J. (2015). Nanogrooved substrate promotes direct lineage reprogramming of fibroblasts to functional induced dopaminergic neurons. *Biomaterials*.

[51] Li Y., Chu Julia S., Kurpinski K. (2011). Biophysical regulation of histone acetylation in mesenchymal stem cells. *Biophysical Journal*.

[52] Morez C., Noseda M., Paiva M. A., Belian E., Schneider M. D., Stevens M. M. (2015). Enhanced efficiency of genetic programming toward cardiomyocyte creation through topographical cues. *Biomaterials*.

[53] Abdeen A. A., Lee J., Li Y., Kilian K. A. (2017). Cytoskeletal priming of mesenchymal stem cells to a medicinal phenotype. *Regenerative Engineering and Translational Medicine*.

[54] Roy B., Venkatachalapathy S., Ratna P. (2018). Laterally confined growth of cells induces nuclear reprogramming in the absence of exogenous biochemical factors. *Proceedings of the National Academy of Sciences*.

[55] Lee J., Molley T. G., Seward C. H. (2020). Geometric regulation of histone state directs melanoma reprogramming. *Communications Biology*.

[56] Provenzano P. P., Eliceiri K. W., Campbell J. M., Inman D. R., White J. G., Keely P. J. (2006). Collagen reorganization at the tumor-stromal interface facilitates local invasion. *BMC Medicine*.

[57] Chaudhuri P. K., Pan C. Q., Low B. C., Lim C. T. (2016). Topography induces differential sensitivity on cancer cell proliferation via rho-ROCK-myosin contractility. *Scientific Reports*.

[58] Beliveau A., Thomas G., Gong J., Wen Q., Jain A. (2016). Aligned nanotopography promotes a migratory state in glioblastoma multiforme tumor cells. *Scientific Reports*.

[59] Mahendra Y., He M., Rouf M. A. (2021). Progress and prospects of mechanotransducers in shear stress-sensitive signaling pathways in association with arteriovenous malformation. *Clinical Biomechanics*.

[60] Vania V., Wang L., Tjakra M. (2020). The interplay of signaling pathway in endothelial cells--matrix stiffness dependency with targeted-therapeutic drugs. *Biochimica et Biophysica Acta (BBA) - Molecular Basis of Disease*.

[61] Wang Y., Wang G., Luo X., Qiu J., Tang C. (2012). Substrate stiffness regulates the proliferation, migration, and differentiation of epidermal cells. *Burns*.

[62] Chen X., Fan Y., Sun J. (2021). Nanoparticle-mediated specific elimination of soft cancer stem cells by targeting low cell stiffness. *Acta Biomaterialia*.

[63] Lei K., Kurum A., Kaynak M. (2021). Cancer-cell stiffening via cholesterol depletion enhances adoptive T-cell immunotherapy. *Nature Biomedical Engineering*.

[64] Li J., Li Y., Gao B. (2018). Engineering mechanical microenvironment of macrophage and its biomedical applications. *Nanomedicine (London, England)*.

[65] Singh S., Awuah D., Rostam H. M. (2017). Unbiased analysis of the impact of micropatterned biomaterials on macrophage behavior provides insights beyond predefined polarization states. *ACS Biomaterials Science & Engineering*.

[66] Chen S., Yang J., Wei Y., Wei X. (2020). Epigenetic regulation of macrophages: from homeostasis maintenance to host defense. *Cellular & Molecular Immunology*.

[67] Lodewijk I., Nunes S. P., Henrique R., Jerónimo C., Dueñas M., Paramio J. M. (2021). Tackling tumor microenvironment through epigenetic tools to improve cancer immunotherapy. *Clinical Epigenetics*.

[68] Kaukonen R., Mai A., Georgiadou M. (2016). Normal stroma suppresses cancer cell proliferation via mechanosensitive regulation of JMJD1a-mediated transcription. *Nature Communications*.

[69] Li Y. F., Tang C. B., Kilian K. A. (2017). Matrix mechanics influence fibroblast-myofibroblast transition by directing the localization of histone deacetylase 4. *Cellular and Molecular Bioengineering*.

[70] You E., Huh Y. H., Kwon A. (2017). SPIN90 depletion and microtubule acetylation mediate stromal fibroblast activation in breast cancer progression. *Cancer Research*.

[71] Kuang W., Tan J., Duan Y. (2009). Cyclic stretch induced miR-146a upregulation delays C2C12 myogenic differentiation through inhibition of Numb. *Biochemical and Biophysical Research Communications*.

